# Cribriform pattern disease over‐represented in pelvic lymph node metastases identified on ^68^GA PSMA‐PET/CT

**DOI:** 10.1002/bco2.151

**Published:** 2022-04-21

**Authors:** Damien Bolton, Anne Hong, Nathan Papa, Marlon Perera, Brian Kelly, Catriona Duncan, David Clouston, Nathan Lawrentschuk

**Affiliations:** ^1^ Department of Urology and University of Melbourne Department of Surgery Austin Health Melbourne Australia; ^2^ Department of Preventive Medicine Monash University Melbourne Australia; ^3^ Urology Service, Department of Surgery Memorial Sloan Kettering Cancer Center New York New York USA; ^4^ Tissupath Melbourne Australia; ^5^ Department of Urology Peter MacCallum Cancer Centre Melbourne Australia

**Keywords:** cribriform pattern, pelvic lymph nodes, prostate cancer, PSMA‐PET

## Abstract

**Objectives:**

To determine whether any specific histologic subtype of prostate cancer was preferentially represented in pelvic lymph node metastases identified on ^68^GA‐PSMA‐PET/CT.

**Subjects and Methods:**

A consecutive series of 66 men with biochemical recurrent prostate cancer was evaluated with ^68^GA‐PSMA‐PET/CT. Where disease was confined to pelvic lymph nodes, patients were offered salvage extended pelvic lymph node dissection. Twenty patients ultimately proceeded to extended bilateral template pelvic lymph node dissection. Lymph node positivity and the histologic subtype of apparent cancer were assessed, as was PSA response to this intervention.

**Results:**

Mean PSA at time of PSMA scanning for patients undergoing lymphadenectomy was 2.49 (*n* = 20, range 0.21–12.0). In 16 of 20 patients, there was evidence of metastatic cribriform pattern prostate cancer in excised nodes (100% cribriform pattern in 11/16). Only four of 20 patients had no evidence of this histologic subtype of disease. PSA response was not related to the presence or proportional amount of cribriform pattern disease identified.

**Conclusions:**

Cribriform pattern adenocarcinoma appears to be the histologic subtype preferentially identified in pelvic lymph nodes on ^68^GA‐PSMA‐PET/CT. The use of PSMA‐PET may be particularly valuable in staging of primary or biochemically recurrent prostate cancer in patients with cribriform pattern disease detected on initial biopsy or radical prostatectomy. Further research is required to further confirm the observed association.

## 1 INTRODUCTION

The principal treatment options for intermediate‐risk localised prostate cancer (PC) includes radical prostatectomy (RP) or pelvic radiotherapy (RT).^1^ Following definitive treatment of PC by RP or RT, PSA should diminish to low or undetectable levels. Biochemical recurrence (BCR) has been defined as the detection of PSA levels in excess of 0.20 ng/ml after RP^2^ or nadir +2 ng/ml following RT.^3^ BCR is not uncommon following treatment of PC with up to 50% of patients displaying this effect.^4^ However, PSA only acts as a marker of occult pathology and has limited ability in differentiating between local, regional and distant disease recurrence.

Until recently, radiological investigation of patients with BCR has been limited by the sensitivity of tests such as bone scanning and computed tomography (CT). These modalities rely on the presence of active bony metastatic disease or nodal disease of sufficient size to be identified on imaging. Novel nuclear medical imaging techniques such as prostate‐specific membrane antigen (PSMA) positron emission tomography (PSMA‐PET) may overcome this limitation. Localisation of disease recurrence has been reported with superior sensitivity and specificity compared with conventional imaging.^5^, ^6^, ^7^, ^8^ Accurate staging of disease burden following BCR may permit patient specific management in this setting, including optimal treatment of oligometastatic disease and potential long‐term BCR‐free survival, as has been previously reported following extended pelvic lymph node (LN) clearance in the setting of PET detected pelvic LN recurrent PC.^9^

We performed extended pelvic LN dissection on a consecutive cohort of men being investigated for BCR following definitive treatment of PC, where PC metastases in LN tissue was detected by ^68^GA‐PSMA‐PET/CT. The histopathologic characteristics of nodal metastases in PSMA‐PET visible pelvic LN were evaluated to determine if any specific histopathologic pattern was more likely to be predictive of PSMA‐PET/CT positivity.

## 2 PATIENTS AND METHODS

**FIGURE 1:**
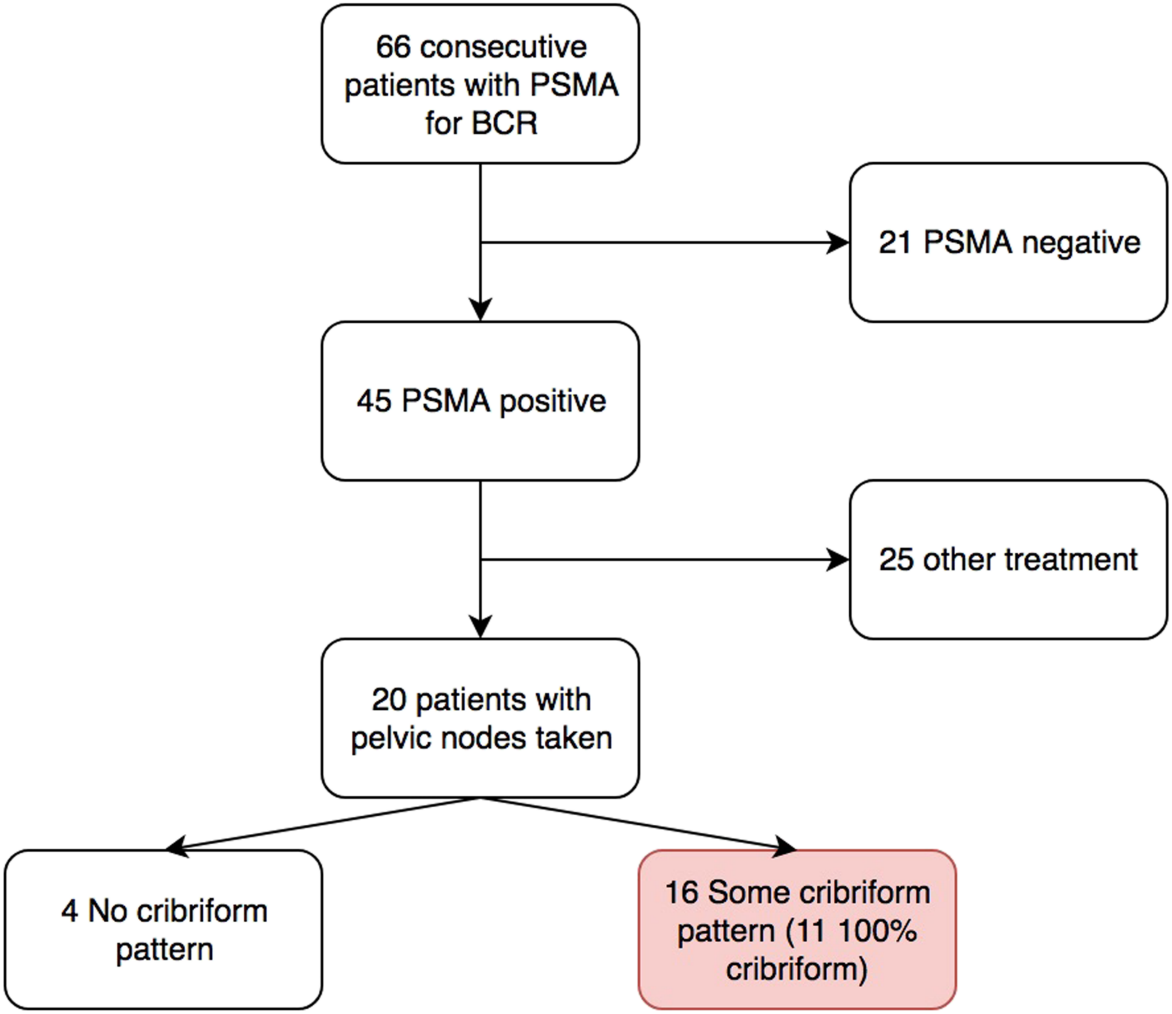
Flow chart of 66 patients investigated for BCR with PSMA‐PET

We retrospectively reviewed 66 men with BCR of PC after earlier radical therapy by either robot‐assisted radical prostatectomy (RARP) or radiation therapy underwent investigation with ^68^GA‐PSMA‐PET/CT (Figure 1). At their initial assessment prior to RP, all men had undergone multi‐parametric magnetic resonance imaging of the prostate, and staging was performed with conventional imaging (computerised tomography and technetium whole‐body bone scan). Patient did not receive pre‐prostatectomy PSMA‐PET; at the time of these cases, PSMA‐PET was only performed in the setting of BCR. In all cases, conventional imaging determines no evidence of metastatic disease, and thus, patients were offered prostatectomy with curative intent. No patient treated by RARP had undergone pelvic LN clearance in association with this primary treatment, consistent with local trends.^10^

The radiopharmaceutical used for the PET study was 68‐gallium radioisotope‐tagged PSMA‐11 ligand (ABX AG, Germany). PET imaging was performed 60 min after intravenous administration of ^68^Ga‐PSMA tracer at a dose of 1.8–2.2 MBq per kilogram. Patients were required to empty their bladder prior to image acquisition. All PET images were collected with a Siemens Biograph mCT 64 slice (Siemens®, Munich, Germany). PET images from the skull vertex to the mid thighs were obtained. CT images were subsequently performed during tidal respiration to allow for lesion localisation. Images were reviewed by a nuclear medicine specialised with considerable experience in urologic radiology and PSMA‐PET. Lesions were considered positive if there was significant uptake, when SUVmax was greater than 2.

Of the patients with PSMA‐positive pelvic nodal disease, a proportion of patients proceeded with salvage extended pelvic LN dissection. All cases were discussed at an institutional multidisciplinary meeting, and cases were managed on grounds of clinical discretion. Patients were offered hormonal deprivation, pelvic irradiation or salvage lymphadenectomy and consented for the ensuing treatment appropriately. Pelvic lymphadenectomy was considered based on previous reports in the setting of oligometastatic disease, resulting in biochemical progression‐free survival of 23%–64% at 2 years.^11^ Delaying BCR also delays initiation of androgen deprivation therapy (ADT).

Extended LN dissection templates were utilised in each case with every patient having bilateral dissection regardless of the PSMA‐PET/CT findings of laterality, with contralateral LN serving as a control study of unilateral PET visible LN. LN samples were taken from perivesical, presacral, obturator, internal iliac, common iliac and external iliac packets and were labelled accordingly for later correlation with PSMA‐PET images.

All lymphadenectomy samples transported to the pathology laboratory in a formalin solution. They were reviewed by a single uropathologist who was blinded to the location of positive LN on the preceding PSMA‐PET study. All LN specimens were fixed by immersion in 10% neutral buffered formalin for at least 24 h. They were embedded with paraffin and then sliced and stained with haematoxylin and eosin. After this, the uropathologist performed histopathological analysis, including the number of LN positive for PC, their size, location, the percentage of nodes positive for metastasis and the histologic subtype of PC noted in the LN. For the cribriform pattern PC, the definition used was ‘A confluent sheet of contiguous malignant epithelial cells with multiple glandular lumina that are easily visible at low power. There should be no intervening stroma or mucin separating individual or fused glandular structures’, in accordance with the International Society of Urological Pathology (ISUP) consensus.^12^

## 3 RESULTS

Following PSMA‐PET/CT, 45 (61.2%) patients had evidence of metastatic deposits identified on imaging. Of these, 19 (42.2%) patients had evidence of disease that had spread beyond regional LN and were consequently treated by ADT. Three patients (6.7%) had evidence of local recurrence in the bed of the previously excised prostate and were treated by salvage RT. Three further patients (6.7%) who had evidence of small‐volume pelvic nodal disease elected to be treated by radiation in a pelvic template. The remaining 20 (44.4%) patients with evidence of PSMA‐PET avid regional LN metastases underwent salvage pelvic LN clearance (16 (80.0%) open, 4 (20.0%) robot assisted), as a means of treatment of their oligometastatic disease, and with intent of deferring introduction of ADT. All patients in this group except one had PET avid LN on one side of the pelvis only.

Of the 20 patients who underwent pelvic LN harvest, 15 (75.0%) had previously received primary treatment by RARP, and five (25.0%) by radiation therapy (three high‐dose‐rate brachytherapy, two external beam radiation [EBRT]). Of the 15 patients that underwent RARP, 7/17 (46%) had extracapsular extension, 4/15 (26.7%) had positive surgical margins, and 9/15 (60.0%) demonstrated cribriform pattern on the prostate histopathology. Of the patients receiving RT, 2/5 (40%) demonstrated cribriform pattern on the diagnostic biopsy. One patient (6.7%) in this subgroup (treated by EBRT) had received 2 years of ADT at the time of original treatment. Five patients (33.3%) who had been treated initially with RARP had also received salvage EBRT to the prostate bed following initial BCR detection.

Mean PSA at time of PSMA scanning for those 20 patients who underwent lymphadenectomy was 2.49 (*n* = 20, range 0.21–12.0), compared with mean PSA 0.4 for the group who had negative PSMA scans (*n* = 21, range 0.1–1.0) and mean PSA of 5.45 for those patients who underwent treatment other than pelvic LN clearance (*n* = 25, range 0.2–19.0 including 19 patients with metastatic disease beyond pelvic LN) (Table 1).

**TABLE 1:** Patient subgroup characteristics

	Some cribriform pattern, *n* = 16	No cribriform pattern, *n* = 4	Other treatment, *n* = 25	PSMA negative, *n* = 21	Total, *n* = 66
Original treatment					
RP	11 (69%)	4 (100%)	21 (84%)	20 (95%)	56 (85%)
RT/Brachy	5 (32%)	0	4 (16%)	1 (5%)	10 (15%)
Time from treatment to PSMA					
0–1 years	7 (44%)	1 (25%)	8 (32%)	5 (24%)	21 (32%)
2–4 years	5 (31%)	2 (50%)	2 (8%)	10 (48%)	19 (29%)
5 + years	4 (25%)	1 (25%)	15 (60%)	6 (29%)	26 (39%)

When histological assessment of the pelvic LN samples was undertaken, evidence of cribriform pattern PC was noted in 16 (80%) of cases. The proportion of metastatic PC that was of cribriform pattern varied between 10% and 100% of the LN tumour volume. In 11/16 (68.8%) cases, 100% cribriform pattern PC was identified in metastatic LN deposits. Differences in LN yield or number of positive LN did not reach statistical significance between groups of patients who did and who did not have evidence of cribriform pattern metastatic disease (Table 2).

**TABLE 2:** Lymph node yield

	Nodes taken. Median (IQR)	Nodes positive. Median (IQR)
Some cribriform pattern	17.5 (11–30)	2 (1–3.5)
No cribriform pattern	18.5 (16–22)	1.5 (1–3)

*Note*: “Some cribriform pattern” ranges in % from 10% to 100% cribriform.

On comparison between histopathologic outcomes and PSMA‐PET findings, all LN with cribriform pattern disease were PSMA‐PET avid. In all cases, there was no metastatic disease found in PSMA non‐avid areas. Specifically, no LN positive for metastatic disease were noted in contralateral LN that had not appeared PET avid on PSMA scanning and that acted as a control group in this study.

**FIGURE 2:**
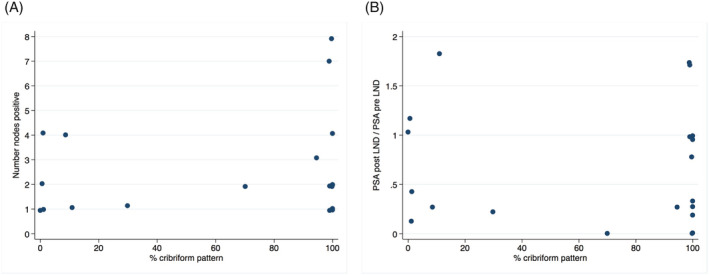
(A) Number of nodes positive by amount of cribriform pattern involvement. (B) PSA response to LND by amount of cribriform pattern involvement

PSA response to treatment by pelvic LN dissection was not related to the presence or proportional amount of cribriform pattern disease identified. PSA was reported as undetectable or <0.02 at 3 months after surgery in only four (20.0%) patients in this series, and seven (35%) patients had PSA levels at or above their level at surgery (Figure 2A,B).

## 4 DISCUSSION

The 68‐gallium labelled ligand of PSMA has established utility in recognising and defining disease recurrence of PC (2). Previous nuclear medicine tracers used in positron emission tomography/computed tomography (PET/CT) have been shown to be less sensitive in detecting lesions in patients with BCR. For this reason, PSMA‐PET/CT is increasingly being incorporated into the clinical algorithm for the investigation of BCR, although subtleties of its function and characterisation are not yet fully established.

The increased sensitivity and specificity of PSMA‐PET/CT compared with conventional imaging give clinicians the ability to detect and locate oligometastatic disease earlier following BCR.^13^ Previously, investigations to localise disease recurrence relied on bone scan and CT findings to suggest metastatic disease. These findings implied an advanced stage that is more likely to be associated with systemic dissemination, leading to the consideration of ADT. One aim of earlier diagnosis of recurrent disease, at the oligometastatic stage, is to delay the introduction of ADT. Earlier detection of disease recurrence has been shown to optimise treatment outcomes in salvage RT^14^ and allows for LN resection to remain as a therapeutic option. Both of these have the potential to achieve an extended period of further BCR‐free survival as has been previously reported in this circumstance.

In our cohort, we have identified an over‐representation of cribriform pattern PC as the dominant architecture of nodal metastatic disease detected by PSMA‐PET. This raises further questions about the nature of PSMA avidity in metastatic nodal disease and appropriate timing in the use of PSMA‐PET. The presence of cribriform pattern subtype on initial pathology has been shown to predict adverse outcomes in men with PC.^15^ Cribriform pattern disease when detected on biopsy has also been displayed as a predictive factor for upstaging of grade on prostatectomy specimen.^16^ With other factors such as PSA kinetics and tumour characteristics (grade group, extra‐capsular extension and seminal vesicle involvement) being taken into consideration for risk stratification, the presence of cribriform pattern may help to identify men at higher risk of clinical progression and should be highlighted in pathology when found. In cases such as these, the value of PSMA‐PET employment earlier in the treatment algorithm should be considered to optimise outcomes.

Compared with normal prostatic glandular tissue, PSMA expression is increased 100‐ to 1000‐fold in dysplastic and neoplastic prostate cells.^17^ This expression has been reported to increase as PC cells progress along the path towards androgen independence.^18^ Differences that exist in the architectural structure of PC have been associated with differing long‐term oncological outcomes.^19^ These types are significant when assessing the risk of progression to BCR and post‐operative metastatic disease and have themselves been identified as prognostic indicators.^20^ Specifically, the presence of cribriform architecture at RP has been associated with a decreased 5‐year BCR‐free survival, metastatic deposits and increased PC‐specific mortality.^15^, ^21^

Cribriform pattern PC was the dominant subtype seen in LN metastases in our patient series. However, as other architectural subtypes of PC were also shown to be PSMA‐PET positive, we must consider the possibility of heterogeneity in PSMA avidity between histopathological PC subtypes. One clear limitation to the current series is the limited sample size. As such, a larger series will be required in future studies to confirm the observed signal. Another limitation to our study is the lack of PSMA‐PET imaging data at the time of initial staging and biopsy. That data could elucidate whether cribriform pattern is more likely than non‐cribriform PC to be represented by PSMA‐PET avidity. Finally, there are several possible selection biases that are relevant for the current cohort, particularly in light of the retrospective nature of the current project. For example, selection biases exist as patients proceeded to salvage nodal dissection based on clinical discretion. Thus, this population is likely not representative of the greater prostatectomy population, which may resultingly impact the prevalence of pathologic patterns. Further, for patients who are healthier and are more likely to undergo surgical management as opposed to pelvic radiation or ADT, this should not affect the finding of cribriform pattern on histology analysis.

In conclusion, cribriform pattern adenocarcinoma was the dominant histologic subtype of PC identified in metastatic disease to pelvic LN on ^68^Ga‐PSMA‐PET/CT. Accordingly, in patients with cribriform pattern disease detected on initial biopsy or RP, the use of PSMA‐PET may be a valuable adjunct in the staging of primary or BCR PC, specifically regarding the presence of micrometastatic disease in pelvic LN. The use of PSMA‐PET to stage this high‐risk group may allow for patient‐tailored therapy approaches, particularly regarding extended LN dissection and determination of primary radiation therapy fields, and possibly identification of response after theranostic intervention. Although the observed trend is of interest, we acknowledge the limited number of patients in the series limits clinical applicability. Further research is required to definitively discern this relationship.
